# Glass Fiber Reinforced Epoxy-Amine Thermosets and Solvate IL: Towards New Composite Polymer Electrolytes for Lithium Battery Applications

**DOI:** 10.3390/ijms241310703

**Published:** 2023-06-27

**Authors:** Natália Magalhães, Beatriz Arouca Maia, Maria Helena Braga, Raquel M. Santos, Nuno Correia, Eunice Cunha

**Affiliations:** 1 Materials and Composite Structures Unit (UMEC), Institute of Science and Innovation in Mechanical and Industrial Engineering (INEGI), 4000-014 Porto, Portugal; nmagalhaes@inegi.up.pt (N.M.); bmaia@inegi.up.pt (B.A.M.); rmsantos@inegi.up.pt (R.M.S.); ncorreia@inegi.up.pt (N.C.); 2 LAETA—Associated Laboratory of Energy, Transports and Aeronautics, 4200-265 Porto, Portugal; mbraga@fe.up.pt; 3 Engineering Physics Department, FEUP—Faculty of Engineering, University of Porto, 4200-265 Porto, Portugal

**Keywords:** composite gel polymer electrolyte, solid state lithium battery, solvated ionic liquid, glass fiber membrane, crosslinked network

## Abstract

To effectively use (Li) lithium metal anodes, it is becoming increasingly necessary to create membranes with high lithium conductivity, electrochemical and thermal stabilities, as well as adequate mechanical properties. Composite gel polymer electrolytes (CGPE) have emerged as a promising strategy, offering improved ionic conductivity and structural performance compared to polymer electrolytes. In this study, a simple and scalable approach was developed to fabricate a crosslinked polyethylene oxide (PEO)-based membrane, comprising two different glass fiber reinforcements, in terms of morphology and thickness. The incorporation of a solvated ionic liquid into the developed membrane enhances the ionic conductivity and reduces flammability in the resulting CGPE. Galvanostatic cycling experiments demonstrate favorable performance of the composite membrane in symmetric Li cells. Furthermore, the CGPE demonstrated electrochemical stability, enabling the cell to cycle continuously for more than 700 h at a temperature of 40 °C without short circuits. When applied in a half-cell configuration with lithium iron phosphate (LFP) cathodes, the composite membrane enabled cycling at different current densities, achieving a discharge capacity of 144 mAh·g^−1^. Overall, the findings obtained in this work highlight the potential of crosslinked PEO-based composite membranes for high-performance Li metal anodes, with enhanced near room temperature conductivity, electrochemical stability, and cycling capability.

## 1. Introduction

Batteries, particularly lithium-based batteries, play a crucial role in achieving the sustainability goals by meeting the increasing demands for mass electrification and efficient energy storage systems. Cutting-edge research has focused on developing new materials for batteries capable of delivering even higher capacity at lower costs, while maintaining safety during operation [1]. However, conventional flammable liquid electrolytes present well-known drawbacks, including dendrite formation that results in decreased cell capacity, as well as heightened risks of short-circuiting, fire, and explosion [2,3].

Gel polymer electrolytes (GPEs), composed of an impregnated polymeric matrix with a liquid plasticizer, are a suitable alternative, since they combine high ionic conductivities, improved chemical stability, and electrode compatibility [4,5]. The incorporation of a liquid plasticizer allows the formation of better electrode interfaces, and results in good cycling stability and improved performance [6,7,8]. However, the liquid plasticizer has a negative impact on the mechanical properties of the final GPEs [9,10]. One of the main strategies to overcome this challenge is based on the incorporation of inorganic materials, resulting in composite gel polymer electrolytes (CGPEs) [11,12]. Glass fibers (GFs) are an ideal reinforcement material for GPEs owing to their exceptional mechanical properties, insulating characteristics, and thermal stability [13,14,15]. Furthermore, the spaces between the GFs in the membrane can be filled with polymer precursor mixtures, facilitating polymerization within them, and leading to the development of a semi-structural composite, where the polymer electrolyte forms the matrix of the composite.

On the other hand, the use of solvated ionic liquids (SILs) is emerging as a good alternative to conventional flammable liquid plasticizers [16,17,18]. SILs consist of equimolar mixtures of Li^+^ salt complexes of lithium bis(trifluoromethanesulfoly)imide (Li[TFSI]) and glymes, including triglyme (G3) and tetraglyme (G4), promoting the formation of a highly coordinated ion gel that shows similar properties to conventional ionic liquids, such as low volatility, high room temperature ionic conductivity, and nonflammability [19]. In addition, SILs are economically attractive, easy to obtain, and prone to high oxidative stability, making their resultant GPEs suitable for Li batteries operating in a wide potential window [20].

Herein, a composite gel polymer electrolyte was developed by ring-opening polymerization of poly(ethylene glycol) diglycidyl ether (PEGDE) with a polyether amine (PEA) in GF three-dimensional (3D) networks. These precursors were carefully selected as crosslinking units, based on their ethylene oxide repeating units, capable of ion solvation. This polymerization route is a favorable strategy for polymer synthesis, since it does not require the use of solvents or catalytic initiators, which can induce side reactions with Li metal in the battery [21,22]. Moreover, this process is easily scalable and cost-effective, boosting industrial applications. Additionally, the crosslinked nature of the membrane confers some mechanical integrity, the precursor mixture was impregnated into a GF membrane to obtain a semi-structural electrolyte. To the best of our knowledge, the use of SIL with amine-epoxy thermoset resins has not been explored as a polymer electrolyte for Li batteries.

Two different types of glass fiber membranes were studied: (i) a glass microfiber GF/A (GFA) with a thickness of 206 µm, and (ii) a plain weave (GFW) with a thickness of 37 µm. The resulted free-standing membranes showed thicknesses of 225 µm (GFA-based) and 70 µm (GFW-based). Upon cell assembly, a CGPE was obtained after impregnation of SIL in the pores of the dry membrane. A controlled amount of SIL was used, to mitigate the expected slight decrease in ultimate tensile strength and elastic modulus after the incorporation of a plasticizer. Moreover, it was possible to prevent the generation of SIL waste, in contrast to conventional methods of fabricating gel polymer electrolytes, which typically involve soaking the dry membrane in a significant quantity of plasticizer, leading to significant wastage [1,23,24].

The results showed that the GFW-based composite electrolyte (CGPE-GFW), after SIL incorporation, could provide high near room temperature ionic conductivity (σ = 1.7 × 10^−4^ S·cm^−1^, at 40 °C). When used in symmetric Li | Li cells, the GFW-based CGPE membrane demonstrated superior stability against Li metal and dendrite formation in comparison to the GFA-based counterpart (CGPE-GFA). Subsequently, when assembled in an LFP | Li half-cell, the CGPE-GFW electrolyte enabled cycling stability at 40 °C, demonstrating the potential application of glass-fiber-reinforced epoxy-amine thermoset polymer composites combined with SIL for energy storage applications.

## 2. Results and Discussion

### 2.1. Morphological Characterization

**Figure 1:**
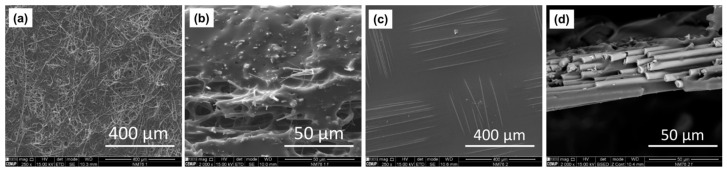
SEM images from CGPE-GFA and CGPE-GFW membranes. (**a**) Surface and (**b**) cross section from CGPE-GFA. (**c**) Surface and (**d**) cross-section from CGPE-GFW.

Scanning electron microscopy (SEM) was used to analyze and compare both the surface and cross-sectional morphology of CGPE membranes before SIL soaking, as shown in Figure 1.

The SEM images show that the surface of the CGPE-GFA membrane (Figure 1a) is more porous than the CGPE-GFW membrane (Figure 1c), although both show a non-uniform pore distribution. A noticeable difference between the two membranes is the extent of fiber exposure at the surface, with fibers in the CGPE-GFA membrane being more exposed compared to those in the CGPE-GFW. Moreover, it is visible that in the CGPE-GFA membrane, fibers are randomly distributed, while the CGPE-GFW fibers are balanced in 0° and 90° directions. Despite these differences, the cross-section visualization of both membranes (Figure 1b,c) demonstrates that, in general, the matrix is well embedded into the fibers, indicating that a good interfacial adhesion was established. A higher amount of polymer matrix impregnated into the CGPE-GFA membrane can be observed (Figure 1b) when compared to the counterpart (Figure 1d), in agreement with the measured thicknesses of both membranes. On the other hand, the lower thickness observed in the CGPE-GFW membrane resulted from the inherent thickness of the glass fiber reinforcement, which, further, impacts the overall dimensions and properties of the developed membrane materials. Moreover, fiber pull-out can be identified on both samples as an important failure mechanism in the composite, indicating poor adhesion between the fibers and polymer electrolyte. This will be addressed in the future through studies on fiber sizing selection.

### 2.2. Chemical Structure Characterization

**Figure 2:**
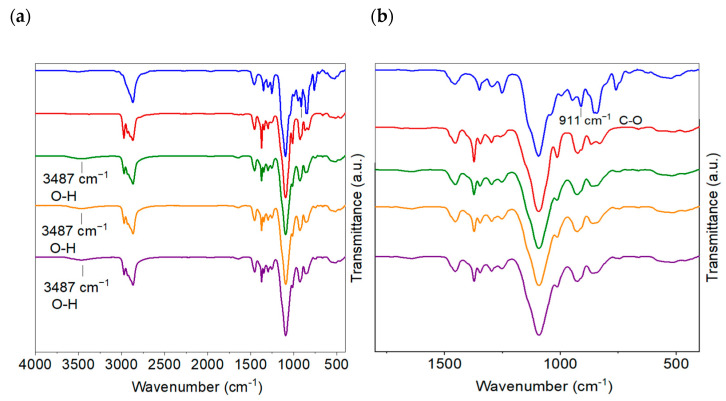
(**a**) ATR-FTIR spectrum of PEGDE (blue), PEA (red), crosslinking matrix (green), and CGPE-GFA (orange) and CGPE-GFW membranes (purple). (**b**) Magnified view of the FTIR spectra between 1800 and 400 cm^−1^.

Attenuated total reflectance Fourier transform infrared spectroscopy (ATR-FTIR) was used to confirm the crosslinking reaction between the epoxy groups of PEGDE and the amine groups of PEA. The ATR-FTIR spectra comparison is summarized in Figure 2.

The spectra of PEGDE and PEA showed the characteristic absorption bands at approximately 2868 and 2970 cm^−1^, attributed to the symmetric and asymmetric stretching vibrations of CH_2_ groups present in the chains of each polymer, respectively [25]. Additionally, strong absorption bands centered at 1100 cm^−1^ are associated with the stretching vibrations of the ether (C–O–C) functional groups derived from both the PEGDE (blue line) and PEA (red line) precursors. The ATR-FTIR spectrum of PEGDE revealed a distinct band at 911 cm^−1^, which can be attributed to the oxirane ring present in the epoxy group [26]. After performing a comparison between the FTIR spectrum of neat epoxy matrix (green line), CGPE-GFA (orange line), and CGPE-GFW (purple line), it was found that this peak disappeared, indicating that the reaction was successfully achieved in free-standing membranes. It is noteworthy that PEA, a related compound, exhibited a similar peak near 911 cm^−1^, although its contribution to the observed spectrum is negligible. As the reaction progressed, the formation of a new peak at 3487 cm^−1^ was also observed, which can be attributed to the hydroxyl (O-H) groups formed during the crosslinking process.

To further evaluate the polymerization degree and obtain a comprehensive understanding of the system, differential scanning calorimetry (DSC) experiments were carried out. This technique combined with thermogravimetric analysis (TGA) provided valuable insights into the thermal transitions and stability of the resulting crosslinked structures.

### 2.3. Thermal Characterization

**Figure 3:**
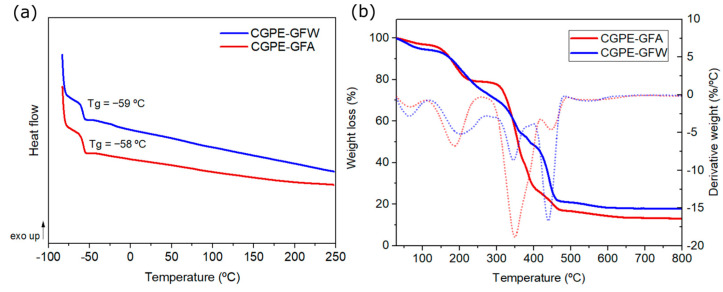
(**a**) DSC thermograms of dry composite electrolytes. (**b**) TGA analysis and its derivative thermogravimetric (DTG) curve (dotted lines) of CGPE-GFA and CGPE-GFW after SIL impregnation.

The thermal behavior of the composite membranes was evaluated, and the results are summarized in Figure 3.

DSC analysis (Figure 3a) confirmed that both composite membranes are completely polymerized, by the absence of residual heat or an exothermal peak, in agreement with previous observations by ATR-FTIR. In addition, negative glass transition temperatures (T_g_) of −58 °C and −59 °C were found for CGPE-GFA and CGPE-GFW, respectively, which, combined with the presence of amorphous phases, are essential for delivering a high ionic conductivity by segmental motion of the ethylene oxide chains above T_g_ [27]. In this case, it is expected that at room temperature both membranes could provide appropriate Li^+^ ion hopping, and a high ionic conductivity.

The thermal stability of the developed membranes was also analyzed by TGA (Figure 3b), and the results revealed that both composite membranes exhibited minimal weight loss up to 155 °C. The CGPE-GFA membrane experienced a weight loss of 3%, while the CGPE-GFW membrane exhibited a weight loss of 5%, due to the release of moisture or incomplete complexation of G4 during the synthesis of the solvated ionic liquid (SIL). These degradation steps can also be identified on the DTG curve. Further analysis showed a second weight loss step, as shown on the DTG curves, for both membranes in the temperature range from 155 °C to 230 °C of approximately 17% and 15% for CGPE-GFA (decomposition temperature, T_d_ = 155 °C) and CGPE-GFW (T_d_ = 162 °C), respectively. This weight loss could be attributed to the volatilization of G4 resulting from the decomplexation of G4 in the [Li(G4)][TFSI] complex. This might be related to the interaction of G4 with the ether moieties from the epoxy resin, leading to some SIL decomplexation [28]. Above 230 °C, a third degradation step was identified (DTG curve), in which the weight loss could be attributed to the decomposition of the SIL complex as well as the epoxy matrix. Overall, both membranes demonstrate that they could be safely used for practical applications near room temperature and below 155 °C, delivering superior thermal stability when compared to traditional flammable liquid electrolytes, which volatilize below 100 °C [29].

### 2.4. Mechanical Characterization

**Figure 4:**
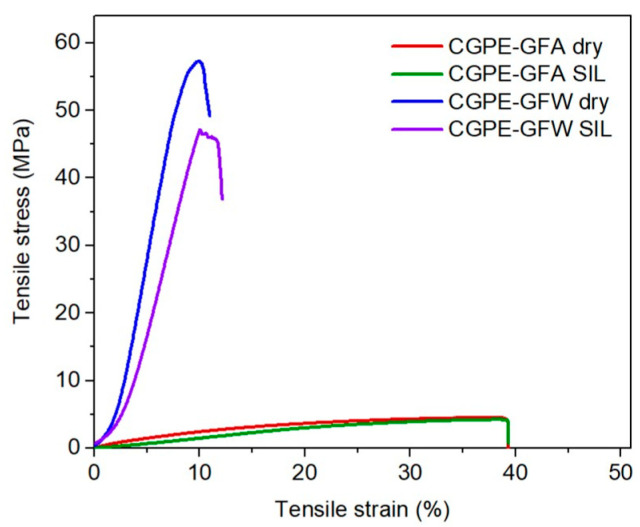
Representative stress–strain curves for dry CGPE-GFA and CGPE-GFW, and CGPE-GFA and CGP-GFW after SIL impregnation.

Stress–strain experiments were performed to evaluate the influence of the SIL incorporation on the mechanical properties of the membranes. In Figure 4, representative curves of both membranes are presented, before and after SIL impregnation.

The CGPE-GFA and CFPE-GFW membranes showed a brittle behavior, without a plastic plateau, due to the high crosslink density of the thermoset polymer. However, the CGPE-GFA membrane presented a lower ultimate tensile strength and elastic modulus in comparison with CGPE-GFW (4.7 vs. 57 MPa, and 34 vs. 111 MPa, respectively), showing the good ability of plain weave glass fibers (balanced in 0° and 90° directions) to reinforce composite membranes, with a good load transfer efficiency. Moreover, the impregnation of SIL in both membranes resulted in a slight decrease in both the Young’s modulus and tensile stress [30], although the CGPE-GFW still maintained fair mechanical properties of a tensile strength of 48 MPa and elastic modulus of 97 MPa, while preserving its tensile strain. Therefore, carefully controlling the amount of plasticizer added can help to maintain a balance between the resultant ionic conductivity and mechanical properties. Apart from that, thicker materials with higher Young’s modulus and tensile strength values typically exhibit lower ionic conductivity, which can be a limiting factor in battery applications [31].

### 2.5. Electrochemical Performance

**Figure 5:**
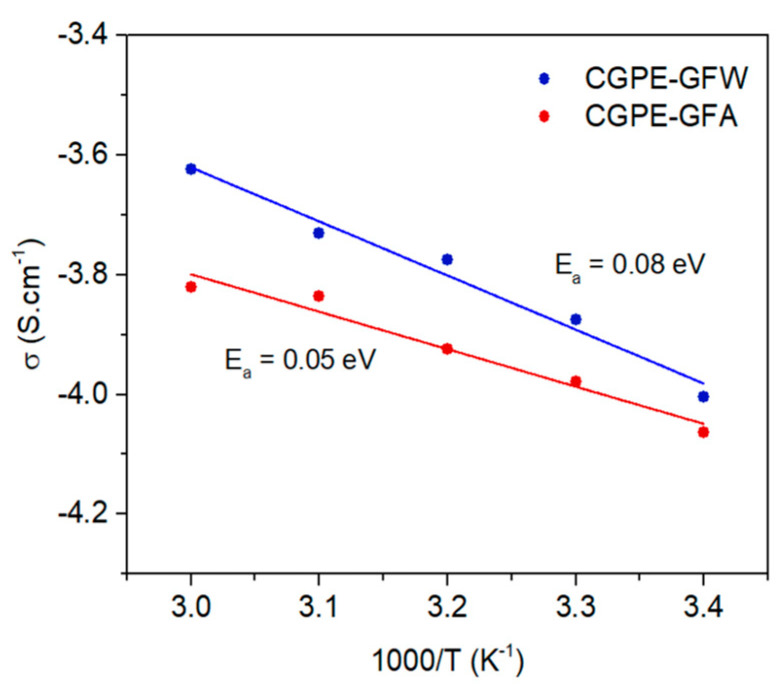
Ionic conductivity and Arrhenius linear fit of CGPE-GFA and CGPE-GFW as a function of temperature.

The effect of temperature on the ionic conductivity, σ, was investigated for both composite electrolyte membranes by means of electrochemical impedance spectroscopy (EIS) on symmetric Cu | Cu cells (Figure 5) and calculated according to Equation (1). For this, dry membranes were allowed to soak with a controlled amount of SIL before the measurements.

According to the results collected by EIS, at 40 °C, both CGPE membranes follow Arrhenius behavior and possess high Li^+^ σ of 1.2 × 10^−4^ S·cm^−1^ and 1.7 × 10^−4^ S·cm^−1^ for CGPE-GFA and CGPE-GFW, respectively, suggesting that they can be suitable for near room temperature energy storage applications. As expected, the ionic conductivity of the composite membranes increased with rising temperature, promoted by the higher segmental motion of polymer chains which facilitated ion hopping mechanisms [32,33]. The CGPE-GFW membrane possesses slightly higher ionic conductivity than the CGPE-GFA membrane, probably due to a reduced thickness and the glass fibers’ distribution/orientation, although the incorporation of SIL plays a major role in the ionic conductivity of the gel composites [34]. The activation energies (E_a_) of the prepared membranes were 0.05 eV and 0.08 eV for CGPE-GFA and CGPE-GFW, respectively, determined from the Arrhenius plots using Equation (2).

**Figure 6:**
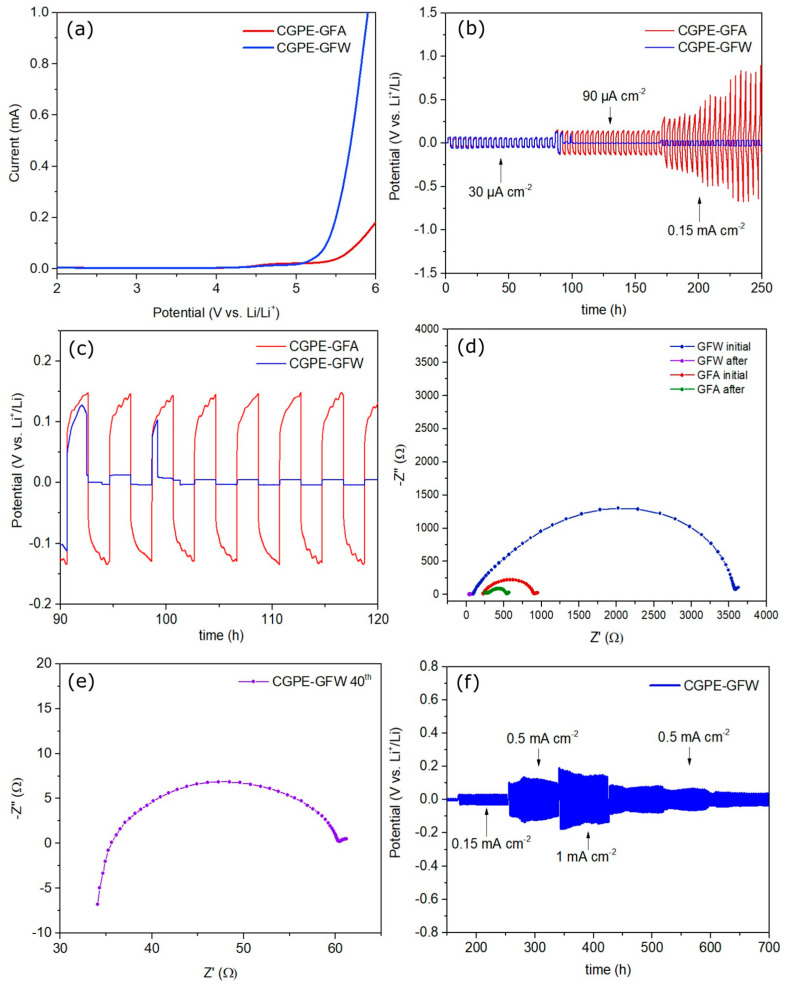
(**a**) LSV plots of the CGPE-GFA and CGPE-GFW Li | Li symmetric cells. (**b**) Galvanostatic Li plating/stripping of CGPE-GFA and CGPE-GFW Li | Li symmetric cells. (**c**) Magnified view of Li plating/stripping of the Li | Li symmetric cells at a current density of 90 µA·cm^−2^. (**d**) EIS spectra of CGPE-GFA and CGPE-GFW Li | Li symmetric cells before and after cycling. (**e**) Magnified view of the EIS spectra of the CGPE-GFW Li | Li symmetric cell after cycling. (**f**) Li plating/stripping performance of CGPE-GFW Li | Li symmetric cell at different density currents.

In addition, the electrochemical stability window of the CGPEs against the Li anode was evaluated, using linear sweep voltammetry (LSV) (Figure 6a).

The anodic voltage stability window is defined as the potential at which a rapid surge in current is observed and continues to rise as the voltage sweep progresses, with the initial current flow corresponding to the electrolyte decomposition [35,36]. It was found that both membranes remained stable up to 5.0 V, indicating that the [Li(G4)]^+^ complex of the SIL is suitable to operate at this voltage, with no formation of free glyme, prone to oxidation [28,37]. The CGPE-GFA membrane exhibited a slightly higher stability of 5.5 V when compared to the CGPE-GFW membrane, which remained stable up to 5.4 V. These findings indicate that both membranes possess a broad electrochemical stability window, making them suitable for use with a wide range of common cathode materials in battery applications [38].

To further evaluate the electrolyte’s compatibility with the Li metal anode, Li | Li symmetric cells were assembled. Galvanostatic cycling tests were then performed to investigate the long-term cycling stability and resistance to dendrite formation. The cells were cycled at 40 °C, at different current densities of 30 µA·cm^−2^, 90 µA·cm^−2^, 0.15 mA·cm^−2^, 0.5 mA·cm^−2^, and 1 mA·cm^−2^, as shown in Figure 6b.

At lower current densities the CGPE-GFA cell delivered overpotentials of 56 mV, increasing to 140 mV with a current density of 90 µA·cm^−2^. The opposite behavior was observed for the CGPE-GFW cell, which initially showed a similar overpotential, at a current of 30 µA cm^−2^, for over 84 h. The magnified view displayed in Figure 6c shows that the interfacial resistance of the CGPE-GFW cell increased its overpotential, and then it lowered and stabilized at only 4.6 mV after 100 h of Li stripping/plating, as a result of a stable Li–electrolyte interface formation [39,40].

To support these results, EIS was performed on both cells, before and after galvanostatic cycling. It was found that the interfacial resistance between the electrolyte and electrode significantly increased after the cell assembly, as can be seen in Figure 6d. Despite having a smaller thickness, the CGPE-GFW membrane showed a pristine higher interfacial resistance than the CGPE-GFA membrane, maybe due to incomplete Li–electrolyte interface formation [41,42]. However, after 100 h of cycling, a large decrease in the interfacial resistance of the cell from 3619 Ω to 61 Ω was observed (Figure 6e), as well as a decrease in the electrolyte’s resistance to 8 Ω. On the other hand, although the CGPE-GFA membrane also reduced its resistance with cycling, from 895 Ω to 537 Ω, it could not stabilize at a lower resistance and reduce its overpotential towards Li metal, possibly due to its higher thickness and GF distribution, compared to the GFW-based membrane. Thus, applying lower current densities in the first cycles seems to be beneficial to the CGPE-GFW membrane, as it may lead to the formation of a stable solid electrolyte interface (SEI) between the electrolyte and Li metal, leading to a decrease in the cell resistance and consequent overpotential during galvanostatic cycling, which allows a continuous stable cycling without short-circuiting the cell [43].

When subjected to a higher current density of 0.15 mA·cm^−2^ the CGPE-GFA membrane displayed an increased overpotential that started at 280 mV reaching 0.9 V after 250 h of cycling, highlighting its inability to endure higher current densities due to excessive resistance. In comparison, the GFW composite membrane only showed a 30 mV overpotential during the same period. When the current density was further increased to 0.5 mA·cm^−2^, the overpotential of the CGPE-GFW membrane continued to cycle with a lower overpotential of 120 mV, as can be seen in Figure 6f. Then, the symmetric cell was subjected to a current density of 1 mA·cm^−2^ and demonstrated stable cycling without exhibiting a high overpotential, reaching only 140 mV after 40 h of cycling at that current density. Moreover, the CGPE-GFW membrane displayed reversibility by lowering its overpotential to 81 mV when the current density was lowered to 0.5 mA·cm^−2^. Interestingly, after continuous cycling, the overpotential gradually decreased and stabilized at 40 mV, performing 700 h of continuous stripping/plating, without showing signs of short-circuiting the cell. This is attributed to the formation of a more conductive interface, which results in a decrease in the polarization resistance [44,45]. Overall, the GFW composite membrane showcased its capability for long-term cycling at higher current densities without the formation of dendrites, making it a more suitable option for applications where high current densities are required [46].

**Figure 7:**
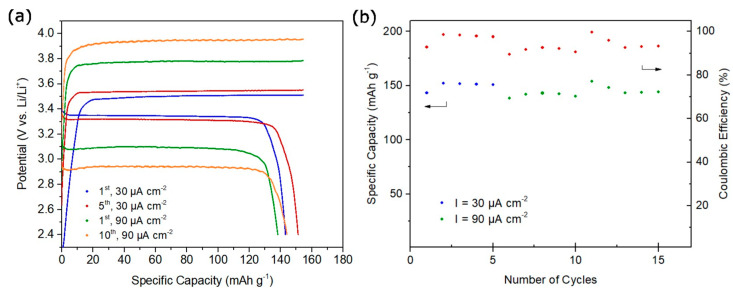
(**a**) CGPE-GFW charge–discharge curves at current density of 30 and 90 µA·cm^−2.^ (**b**) CGPE-GFW rate performance. Blue and green points refer to specific capacity and red points refer to coulombic efficiency.

The cycling performance of an LFP | CGPE-GFW | Li half-cell was tested to evaluate its practical applicability, as shown in Figure 7.

The cell was cycled at 40 °C, under different current densities of 30 µA·cm^−2^ and 90 µA·cm^−2^, between 2.4 V and 4.0 V, or until a maximum capacity of 155 mAh·g^−1^ was reached. Initially, the cells underwent five conditioning cycles at 30 µA cm^−2^, similar to the Li | Li symmetric cells. Figure 7a shows the obtained charge–discharge curves. At 30 µA·cm^−2^ the cell delivered a discharge capacity of 143 mAh·g^−1^ for the first cycle, which is close to the reported specific capacity (155 mAh·g^−1^), with a 93% coulombic efficiency (Figure 7b). Over the cycling, the cell performance gradually improved to a 151 mAh·g^−1^ discharge capacity and 98% coulombic efficiency. After increasing the current density to 90 µA·cm^−2^ the cell showed a drop in the coulombic efficiency as well as discharge capacity, possibly due to reconditioning of the cell to higher current densities. With continuous cycling, the cell improved its discharge capacity as well as coulombic efficiency, showing for its 10th cycle 144 mAh·g^−1^ and 93%, respectively.

The primary aim of this study is to showcase the advantages of utilizing epoxy-amine chemistry in conjunction with solvated ionic liquid (SIL) to create innovative composite gel polymer electrolytes. It should be noted that additional refinements, such as optimizing the cathode design and interfaces, are necessary to enhance the cycling performance even further, as the battery performance test was carried out using commercially available LFP cathode foils.

## 3. Materials and Methods

### 3.1. Materials

Poly(ethylene glycol) diglycidyl ether (PEGDE, Mn ~500), and poly(propylene glycol) bis(2-aminopropyl ether) (PEA, Mn ~2000) were obtained from Aldrich (Schnelldorf, Germany). Lithium bis(trifluoromethanesulfonyl)imide (LiTFSI, 99.95%), tetraglyme (G4, 99%), and acetone (HPLC grade) were obtained from Fisher (Illkirch, France). The G4 and LiTFSI were stored under inert conditions. An MTI (Richmond, USA) Li-ion battery aluminum foil single-side-coated LFP cathode was purchased (reported specific capacity = 155 mAh·g^−1^, 83 µm thickness, active material density = 11.16 mg·cm^−2^). All reagents were used as received without further purification.

### 3.2. SIL [Li(G4)][TFSI] Preparation

As described elsewhere [19], equimolar amounts of LiTFSI and G4 were stirred overnight under an inert atmosphere at 70 °C to form the [Li(G4)][TFSI] complex.

### 3.3. GPE Dry Membrane Preparation

**Figure 8:**
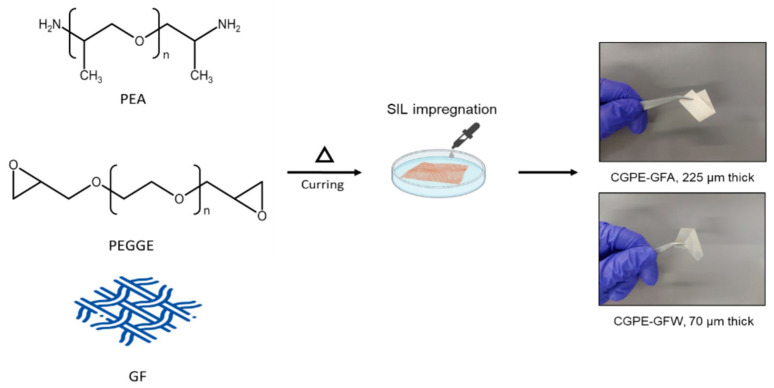
Schematic illustration of the preparation of the composite polymer membrane and composite gel polymer electrolyte with photographs of the free-standing membranes.

A crosslinked PEO membrane was synthesized by amine-epoxide ring-opening polymerization (Figure 8). PEGDE and PEA were dissolved in acetone and stirred at an amine:epoxy ratio of 1:1 to form a homogeneous precursor solution. A certain amount of the precursor solution was impregnated in GFA or glass fiber weave and transferred to a silicone mold and heated at 120 °C for 4 h to obtain a free-standing membrane with 225 µm or 70 µm thickness, for GFA and GFW, respectively.

### 3.4. Coin Cell Assembly and GPE Formation

The 2032-type coin cells were prepared for electrochemical testing. The cells were assembled in an Ar-filled glovebox (O_2_ < 3 ppm, H_2_O < 0.8 ppm) The cells were assembled by placing the LFP cathode (15 mm diameter) on the positive case, followed by a GPE dry membrane (18 mm diameter). A volume of 70 µL of SIL was dropped over the GPE to allow the membrane to soak in the SIL. After that, Li foil (10 mm diameter) was placed over the GPE, followed by a Cu current collector, spacer, spring, and negative case to form the coin cell ready to be closed by hydraulic press.

### 3.5. Characterization

Differential scanning calorimetry (DSC) measurements were performed using a TA instrument, Q20 series (TA instruments, New Castle DE, United States). DSC scans were run between −80 °C and 250 °C, at a scan rate of 5 °C·min^−1^, under a nitrogen flow of 50 mL·min^−1^.

Fourier transform infrared (FTIR) spectra were recorded with an FTIR Perkin Elmer Spectrum Two spectrometer equipped with a diamond crystal (ATR) (Perkin Elmer, Shelton, United States). The samples were used directly without further preparation. The IR spectra were recorded by accumulation of 32 scans at a 4 cm^−1^ spectral resolution over the range from 400 to 4000 cm^−1^ with background subtraction.

The morphology and structural information of the GPEs was collected using a high-resolution (Schottky) environmental scanning electron microscope with X-ray microanalysis: FEI Quanta 400 FEG ESEM (Thermo Fisher, Massachusetts, United States).

Samples were coated with a gold/palladium (Au/Pd) thin film by sputtering, using the SPI module sputter coater equipment.

The thermal properties were characterized by thermogravimetric analysis (TGA) using an STA 449 F3 Jupiter NETZSCH (Selb, Germany). A temperature sweep from 30 °C to 800 °C was conducted with a heating rate of 10 K·min^−1^, under a nitrogen flow.

Mechanical testing was performed on an electromechanical INSTRON (Instron, Norwood, MA, USA) 5900R tensile testing machine and a load cell of ±1 kN. The tensile tests were displacement-controlled with a rate of 1 mm·min^−1^.

### 3.6. Electrochemical Measurements

The electrochemical performance of the developed GPE and correspondent cells were evaluated from the subsequent electrochemical tests.

Electrochemical impedance spectroscopy (EIS) was performed using the Gamry reference 3000 potentiostat/galvanostat/ZRA (Gamry, Warminster, United States). Full cell charge–discharge experiments and cronopotentiometry (CP) with the symmetric cells’ tests were conducted using a Neware CT-4008Tn-5v20mA-164 battery cycler (Neware, Shenzhen, China).

EIS analyses were performed to determine the ionic conductivity of the GPE membranes using an AC current with an amplitude of ±10 mV in the frequency range from 1 MHz to 100 mHz, from 20 °C to 60 °C. The samples were equilibrated at the determined temperature for 1 h between each measurement. The GPE membranes were placed between two copper electrodes. The bulk resistance of the membranes was used to calculate the conductivity, by fitting the equivalent circuits of the resultant Nyquist plots. The ionic conductivity was calculated using Equation (1)

σ = d/AR,(1)

where σ represents the ionic conductivity, d the thickness of the electrolyte, A the area of the electrode, and R the bulk resistance of the electrolyte. The Arrhenius equation (Equation (2))

σ = Aexp[−E_a_/RT], (2)

where A is a pre-exponential factor constant, E_a_ is the activation energy (eV), R is the universal gas constant (J·K^−1^·mol^−1^), and T is the temperature (K), was applied for the determination of the activation energy for ionic mobility in the prepared membranes in the tested temperature range.

The Li symmetric cells were used to evaluate the Li stripping/plating stability at various current densities (30 µA·cm^−2^, 90 µA·cm^−2^, 0.15 mA·cm^−2^, 0.5 mA·cm^−2^, and 1 mA·cm^−2^). Each galvanostatic stripping and plating was maintained for 2 h. All cells were equilibrated at the determined temperature for 1 h between each measurement.

The cycling experiments of the LFP | Li half-cells were carried out between 2.4 and 4.0 V using a series of current densities of 30 µA·cm^−2^ and 90 µA·cm^−2^. The cells were preconditioned for 5 cycles at 30 µA·cm^−2^ before each test. During the charge cycle, a constant current was applied first, until the upper cutoff voltage was reached, then a constant voltage was applied, until the reported specific capacity (155 mAh·g^−1^) was reached or I < 5 µA. Discharge cycles were carried out using a constant current. Between each charge–discharge cycle, there was a rest time of 10 min.

## 4. Conclusions

Significant progress has been shown in the successful development of glass-fiber-reinforced epoxy-amine composites through a simple crosslinking reaction. The incorporation of nonflammable SIL in the membranes did not compromise their mechanical integrity, thanks to the reinforcement provided by the glass fibers. Notably, the CGPE-GFW membrane exhibited superior mechanical performance compared to the others, namely, 48 MPa of tensile strength and 97 MPa Young’s modulus, after SIL impregnation. The developed membranes also demonstrated high ionic conductivity near room temperature, with CGPE-GFW exhibiting particularly remarkable conductivity of 1.7 × 10^−4^ S·cm^−1^ at 40 °C and a wide electrochemical window of 5.4 V. Furthermore, when assembled in Li | Li symmetric cells, the CGPE-GFW membrane exhibited outstanding galvanostatic stripping/plating behavior, showing reduced interfacial resistance even under higher current densities, with stable operation for more than 700 h. Additionally, when assembled in an LFP | Li cell, the CGPE-GFW membrane showcased cycling capability at different current densities, along with high coulombic efficiency. Preliminary results at a current density of 90 µA·cm^−2^ showed that the cell delivered 144 mAh·g^−1^ of discharge capacity and 93% coulombic efficiency, after 10 cycles. Overall, this work presents a simple and scalable strategy for preparing safer composite polymer electrolytes suitable for energy storage applications near room temperature.

## Data Availability

Not applicable.
